# Cohort profile: seek, test, treat and retain United States criminal justice cohort

**DOI:** 10.1186/s13011-017-0107-4

**Published:** 2017-05-16

**Authors:** Redonna Chandler, Michael S. Gordon, Bridget Kruszka, Lauren N. Strand, Frederick L. Altice, Curt G. Beckwith, Mary L. Biggs, William Cunningham, J.A. Chris Delaney, Patrick M. Flynn, Carol E. Golin, Kevin Knight, Alex H. Kral, Irene Kuo, Jennifer Lorvick, Robin M. Nance, Lawrence J. Ouellet, Josiah D. Rich, Stanley Sacks, David Seal, Anne Spaulding, Sandra A. Springer, Faye Taxman, David Wohl, Jeremy D. Young, Rebekah Young, Heidi M Crane

**Affiliations:** 1 Division of Clinical Innovation, National Center for Advancing Translational Sciences, National Institutes of Health, Washington, DC USA; 2 Friends Research Institute, Baltimore, MD USA; 3 Department of Biostatistics, University of Washington, Seattle, WA USA; 4 Medicine and Epidemiology, Yale School of Medicine, New Haven, CT USA; 5 Division of Infectious Diseases, Alpert Medical School of Brown University, Providence, RI USA; 6 Department of Health Policy and Management, Medicine, General Internal Medicine, University of California at Los Angeles, Los Angeles, CA USA; 7 Department of Epidemiology, University of Washington, Seattle, WA USA; 8 Texas Christian University, Fort Worth, TX USA; 9 Departments of Health Behavior and Medicine, University of North Carolina, Chapel Hill, NC USA; 10 Institute of Behavior Research, Texas Christian University, Fort Worth, TX USA; 11 Urban Health Program, RTI International, Research Triangle Park, NC USA; 12 Department of Epidemiology and Biostatistics, The George Washington University, Washington, DC USA; 13 Department of Medicine, University of Washington, Seattle, WA USA; 14 Department of Epidemiology, University of Illinois at Chicago, Chicago, IL USA; 15 Medicine and Epidemiology, Brown University, Center for Prisoner Health and Human Rights, The Miriam Hospital, Providence, RI USA; 16 Center for the Integration of Research & Practice (CIRP), National Development & Research Institutes, Inc. (NDRI), New York, NY USA; 17 Department of Global Community Health and Behavioral Sciences, Tulane University School of Public Health and Tropical Medicine, New Orleans, LA USA; 18 Department of Epidemiology, Emory University, Atlanta, GA USA; 19 Department of Internal Medicine, Yale School of Medicine, New Haven, CT USA; 20 Department of Criminology, Law and Society, George Mason University, Fairfax, VA USA; 21 School of Medicine, University of North Carolina, Chapel Hill, NC USA; 22 Infectious Disease Fellowship Program, University of Illinois at Chicago, Chicago, USA; 23 School of Medicine, University of Washington, Seattle, WA USA

**Keywords:** Criminal justice, HIV, Data harmonization.

## Abstract

**Background:**

The STTR treatment cascade provides a framework for research aimed at improving the delivery of services, care and outcomes of PLWH. The development of effective approaches to increase HIV diagnoses and engage PLWH in subsequent steps of the treatment cascade could lead to earlier and sustained ART treatment resulting in viral suppression. There is an unmet need for research applying the treatment cascade to improve outcomes for those with criminal justice involvement.

**Methods:**

The Seek, Test, Treat, and Retain (STTR) criminal justice (CJ) cohort combines data from 11 studies across the HIV treatment cascade that focused on persons involved in the criminal justice system, often but not exclusively for reasons related to substance use. The studies were conducted in a variety of CJ settings and collected information across 11 pre-selected domains: demographic characteristics, CJ involvement, HIV risk behaviors, HIV and/or Hepatitis C infections, laboratory measures of CD4 T-cell count (CD4) and HIV RNA viral load (VL), mental illness, health related quality of life (QoL), socioeconomic status, health care access, substance use, and social support.

**Results:**

The STTR CJ cohort includes data on 11,070 individuals with and without HIV infection who range in age from 18 to 77 years, with a median age at baseline of 37 years. The cohort reflects racial, ethnic and gender distributions in the U.S. CJ system, and 64% of participants are African-American, 12% are Hispanic and 83% are men. Cohort members reported a wide range of HIV risk behaviors including history of injection drug use and, among those who reported on pre-incarceration sexual behaviors, the prevalence of unprotected sexual intercourse ranged across studies from 4% to 79%. Across all studies, 53% percent of the STTR CJ cohort reported recent polysubstance use.

**Conclusions:**

The STTR CJ cohort is comprised of participants from a wide range of CJ settings including jail, prison, and community supervision who report considerable diversity in their characteristics and behavioral practices. We have developed harmonized measures, where feasible, to improve the integration of these studies together to answer questions that cannot otherwise be addressed.

## Background

### STTR treatment cascade

The Seek, Test, Treat and Retain (STTR) treatment or HIV care cascade is a challenging yet potentially beneficial response to addressing HIV in an era of effective treatments [1–3]. This approach requires reaching out to at-risk individuals who have not been tested for HIV recently (Seek), engaging them in HIV testing (Test), initiating persons living with HIV (PLWH) on antiretroviral therapy (ART) and other treatment services (Treat), and facilitating uninterrupted HIV care (Retain) [1, 2]. The STTR treatment cascade provides a framework for research aimed at improving the delivery of services, care and outcomes of PLWH. There is substantial dropout across each cascade step, and it has been estimated that only ~19% of PLWH in the United States (US) are aware of their HIV diagnosis, engaged in care, on ART, and have an undetectable viral load (VL) [1], although more recent numbers suggest improvements [4, 5]. The development of effective approaches to increase HIV diagnoses and engage PLWH in subsequent steps of the treatment cascade could lead to earlier and sustained ART treatment resulting in viral suppression. Improvements in the STTR treatment cascade have the potential to benefit the health of PLWH and improve public health by reducing HIV transmission [2].

### The relevance of understanding the STTR treatment cascade across all CJ settings

In the US, there are two main types of correctional facilities—jails, that typically are administered by city or county governments, and hold people awaiting trials or serving shorter sentences (generally under two years), and prisons that typically are administered by state and federal governments and hold people serving longer sentences or who have had their parole or probation revoked. Community supervision includes pretrial, probation and parole. Pretrial refers to people awaiting trial before receiving a criminal conviction or acquittal. Probation refers to adults who have been placed on supervision in the community, typically through local or state court systems; about half of individuals on probation also serve a short jail sentence. Parole refers to people who are released early from prison to serve the remaining part of their sentence in the community [6]. The criminal histories of people in prison typically are more serious, lengthy, and varied than individuals in jail or on community supervision [7, 8].

Currently, >95% of incarcerated individuals will be released and re-enter society with nearly 80% being released to parole supervision [9]. About 1 in 35 adults in the U.S. are under CJ supervision, and the number is expected to continue to increase [10]. This trend expands opportunities for HIV prevention and treatment, especially in the public health realm [11]. Specifically, the estimated HIV prevalence among individuals incarcerated in the US prison system is 2-3 times higher than the general population [12–16]. These statistics translate to one in seven PLWH being incarcerated each year, a figure that rises to one in five PLWH who are African-American or Hispanic [17]. Although HIV testing rates in federal and state prisons are generally high (71%), [18, 19] testing rates in jails are not (19%) [18]. Because many CJ-involved individuals pass only through jail, it is likely that many who are infected will not be offered HIV testing [18, 20]. These individuals may not know their HIV status or their potential to transmit HIV [21]. Lack of testing among this higher risk group, and early ART initiation for those who are infected, is a lost opportunity to improve individual and public health and decrease HIV transmission. Those with HIV who return to the communities where they may lack access to health care services, including screening and early diagnosis, are likely to receive HIV treatment only after the disease has progressed to advanced stages [16, 22]. As such, the Centers for Disease Control and Prevention (CDC) encourages more frequent HIV testing of persons within the CJ system, particularly those with drug-dependencies, because PLWH who learn their HIV status are less likely to spread HIV and more likely to seek medical treatment that can lower the potential for HIV transmission and reduce HIV-associated morbidity and mortality [23, 24]. Finally, while PLWH incarcerated in prisons have access to care, including ART, maintaining ART and other medical services is a significant challenge for many re-entering the community following incarceration in prison [25] and engagement in the HIV care cascade has been shown to decline substantially after release [26]. Effective interventions are essential to link individuals to appropriate post-incarceration care and enhance retention in HIV care. The CJ involvement makes this a particularly unique and important cohort for addressing key questions across the HIV treatment cascade.

## Methods

### Cohort development

The STTR CJ cohort is the product of the STTR Data Collection and Harmonization Initiative developed by the National Institute of Allergy and Infectious Diseases (NIAID), the National Institute on Drug Abuse (NIDA), the National Institute of Mental Health (NIMH), and the Office of AIDS Research (OAR). This cohort was developed by a collaboration with researchers who harmonized and pooled data from independent prospective research studies funded under a grant mechanism focused on enhancing the STTR treatment cascade for individuals involved with the CJ system across the US [27]. This initiative produced another cohort not described here focused on vulnerable populations particularly substance users at risk for or with HIV in international and domestic settings. While the STTR initiative and its rationale have been previously described [27], the cohort itself has not yet been described.

### Cohort management structure

The STTR CJ collaboration consists of a three way partnership between 1) individual researcher teams responsible for conducting the STTR studies and overseeing all enrollment and data collection; 2) a scientific officer and program officials from NIAID, NIDA, NIMH, and OAR who provide expertise and guidance regarding the data harmonization initiative; and 3) the STTR Data Coordination Center (DCC), which provides expertise in data harmonization, epidemiology, biostatistics, and HIV, harmonizes the data, supports investigations that use the harmonized data to answer novel scientific questions, and develops new methods for analysis of complex pooled data.

### Cohort description

The STTR CJ cohort is unique in its focus on data from individuals involved in the CJ system and includes 11 NIAID, NIDA, NIMH, and OAR funded studies, several of which involve multiple sub-components and embedded studies. It includes single and multi-site randomized controlled and observational trials evaluating interventions to enhance care delivery along the HIV treatment cascade. It provides a large sample across CJ settings (jail, prison, community supervision) and includes both PLWH and HIV-uninfected individuals, many of whom engage in illicit drug use. Compared to single studies, the CJ cohort data provide increased statistical power to determine public health benefits of the STTR paradigm, address research questions on specific groups at risk of exiting the treatment cascade, and improve understanding of the intersection of drug use and HIV treatment. This cohort profile describes data from the resulting cohort and individual CJ studies including baseline demographic and clinical characteristics.

### Data harmonization

**Fig. 1:**
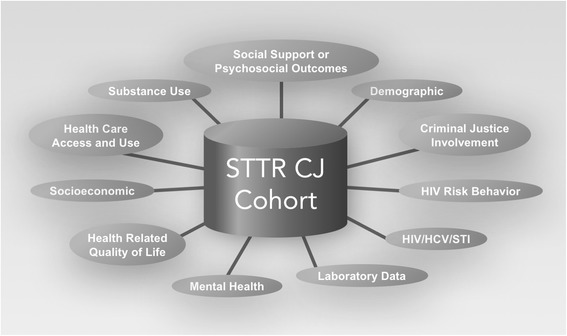
STTR CJ domains selected for data harmonization

Eleven domains were selected for data harmonization: CJ involvement; HIV risk behaviors; HIV and/or hepatitis C infection; laboratory measures of CD4+ cell count and HIV RNA viral load among those with HIV; mental health; health related quality of life; socioeconomic; health care access; substance use and alcohol use; and social support (Fig. 1). The goal is to integrate data from across studies to address research questions requiring sample sizes beyond that of a single study.

The DCC, with investigators from individual studies, is responsible for data integration. The DCC provides technical support and oversees a web-based project collaboration and document management system; documents procedures for data collection and translation, transmission, and quality control; develops integration methods; and generates analyzable data sets. Studies typically upload data biannually. The DCC coordinates data integration from studies sometimes collected using different instruments or timeframes to ensure that the data are comparable across studies in meaning and content. To accomplish this, an examination of data types collected by each study was conducted, working closely with individual study personnel to survey each study’s protocol including how data were collected and modifications to the instruments used. Where data were comparable, codes were developed to create standardized sets of variables.

### Availability of data and materials

The datasets generated during the current study are not publically available due to privacy/license concerns. However, we welcome collaboration from interested parties. We have policies to ensure multi-center analytic research proposals are developed and results analyzed collaboratively and fairly. We are guided by principles of other cohort collaborations [28–30] including that data at the DCC will be stripped of all protected health information (PHI); an individual study can choose to participate or not in any scientific aim or sub-aim; and concept proposals and completed manuscripts must be approved by the Publications and Presentations (P&P) committee. We appreciate and encourage mentorship and the use of the STTR data to enable early investigators to address meaningful questions with support to help ensure their success. Additional information can be obtained at the STTR website: https://sttr-hiv.org/cms or by contacting the STTR DCC at STTR@uw.edu.

## Results

### Description of the STTR CJ studies and participants

**Fig. 2:**
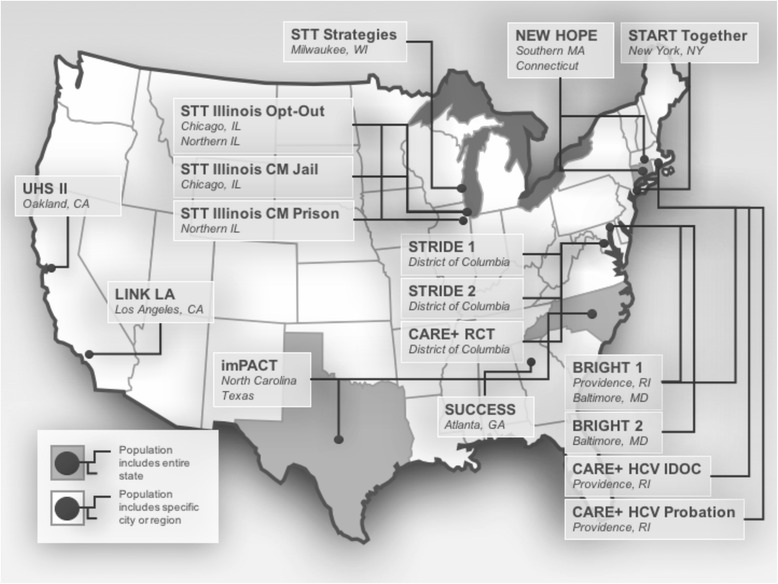
STTR CJ participating sites

Figure 2 shows the diverse geography of the participating studies. For each of the 11 studies and their substudies, the research topic, the components of the STTR treatment cascade addressed, and the targeted participants are described in Table 1. All the steps of the treatment cascade are included in multiple studies, and a diverse array of research topics are included. The STTR CJ cohort includes studies focused on later steps in the STTR treatment cascade such as treatment and retention of PLWH and studies focused on earlier steps such as HIV testing. Therefore, the percentage of PLWH in the individual STTR CJ studies at baseline varies, ranging from 0 to 100% (Table 1), with an overall baseline total of 1888 PLWH. For each of the 11 studies and their substudies, detailed inclusion and exclusion criteria, and the study design including whether it was observational or a trial is described in Table 2. In addition to baseline information, most studies collected data at 3 and/or 6-month follow-up time points, and many include 12 month and other follow-up time points (Table 2).

**Table 1:** Description of STTR CJ cohort studies including research topics, targeted study participants, and HIV status of participants

Study	Enrollment start date	Treatment cascade	Research topic	Targeted study participants	HIV, %
BRIGHT					
BRIGHT 1	2011	S, T	HIV testing	On probation or parole	0.1
BRIGHT 2	2011	Tr, R	Linkage to HIV care via intensive CM	HIV-infected; on probation or parole	100
CARE+					
RCT	2013	Tr, R	Linkage to HIV care via multi-component intervention	HIV-infected; in jail or recently released	100
HCV RIDOC	2012	S, T	Rapid HCV testing using pre-test video counseling	Short-term detainees; unknown HCV status	N/A
HCV PROB	2014	S, T	Rapid HCV testing using pre-test video counseling	On probation or parole; unknown HCV status	N/A
IMPACT	2012	Tr, R	Linkage to HIV care and engagement in clinical care via multi-component intervention	HIV-infected inmates; RNA levels <400 copies/mL, on ART; 3 months prior to prison release	100
LINK LA	2012	Tr, R	Linkage to HIV care via peer-based intervention	HIV-infected; in jail	100
NEW HOPE	2011	Tr, R	Medication assisted treatment for substance abuse	HIV-infected; opiate dependent; leaving prison & jail	100
STT	2013	S, T, Tr, R	HIV testing and linkage to HIV care	Jail detainees	0.4
STT ILLINOIS					
OPT OUT	2012	S, T	HIV testing	In jail	N/A
CM JAIL	2013	Tr, R	Linkage to, and retention in HIV care via transition CM	HIV-infected; leaving jail	100
CM PRISON	2013	Tr, R	Linkage to, and retention in HIV care via transition CM	HIV-infected; recently released from prison	100
START	2011	S, T	HIV testing and risk reduction	Substance users entering drug treatment program	N/A
STRIDE					
STRIDE 1	2011	Tr, R	Medication assisted treatment for substance abuse	HIV-infected; opiate dependent	100
STRIDE 2	2014	Tr, R	Medication assisted treatment for substance abuse	HIV-infected; opiate dependent	100
SUCCESS	2014	Tr, R	Linkage to, and retention in, HIV care via intensive CM	HIV-infected; jail detainees	100
UHS II					
UHS IIC	2011	S, T	HIV testing	People who inject drugs or smoke crack cocaine	3.3
UHS IIL	2011	Tr, R	Linkage to and engagement in HIV care via CM	People who inject drugs or smoke crack cocaine and have HIV	100

*ART* antiretroviral therapy, *CJ* criminal justice, *CM* case management, *N/A* not applicable, *HCV* hepatitis C virus, *HIV* human immunodeficiency virus, *R* retain, *RNA* ribonucleic acid, *S* seek, *STTR* seek, test, treat and retain, *T* test, *Tr* treat, *US* United States

**Table 2:** Study design, follow-up, and participant criteria for STTR CJ cohort studies

Study^a^	Study design	Follow-up time points	Individual inclusion and exclusion criteria
BRIGHT			
BRIGHT 1	Randomized/observational study of on-site rapid HIV testing at probation/parole office vs. off-site referral at a community health center	Baseline only	Unknown HIV status; on probation or parole in Baltimore, MD or Providence/ Pawtucket, RI and residence in the Baltimore or Providence/Pawtucket area throughout the study period
BRIGHT 2	RCT of case management (Project Bridge) vs. TAU	3,6,12,15,18 months	HIV-infected; on probation or parole in Baltimore, MD and planned residence in greater Baltimore area throughout the study period
CARE+			
RCT	RCT of CARE+ Corrections intervention	Monthly for 24 months	Aged 18+; HIV-infected; released from the correctional facility or half-way house ≤6 months ago and living in Washington, DC metropolitan community (not a restricted setting, e.g. half-way house) or currently detained in jail with anticipated release to community (not a restricted setting); reading at 8th grade level and English-speaking
HCV RIDOC	Cross-sectional study of HCV testing	Baseline only	Aged 18+; self-reported as HCV negative and documented HCV infection during Department of Corrections time with anticipated release between 3 and 12 weeks from enrollment; English-speaking
HCV PROB	Cross-sectional study of HCV testing	Baseline only	Aged 18+; self-reported as HCV negative or unknown; on probation or parole; English speaking
IMPACT	RCT of IMPACT intervention vs. standard of care	2, 6, 14, and 24 weeks	Aged 18+; HIV-infected with HIV RNA < 400 copies/mL receiving ART who were incarcerated in NC or TX and 3 months prior to release and not convicted of sexual assault, death or serious injury; English-speaking
LINK LA	RCT of intervention	2, 6, 12 months	Male or transgender individuals, aged 18+; HIV infected; incarcerated in a single facility for 5+ days; residing in Los Angeles County, CA upon release; English fluency
NEW HOPE	Placebo-controlled RCT of. extended-release naltrexone	Monthly for 12 months	Aged 18+, HIV-infected, meeting DSM-IV criteria for opioid dependence, within CT corrections system and not pending trial for a felony, within 30 days of being released to greater New Haven, Hartford, Waterbury or Springfield areas or 30 days after release; English- or Spanish-speaking, no liver failure or grade IV hepatitis, no active opioid withdrawal, no receipt of methadone or buprenorphine/naloxone for treatment of opioid dependency, no participation in pharmacotherapy trial in the previous 30 days
STT	Observational study of comprehensive Seek Test Treat strategies with medical record linkage 2 years post-release	2 years	Aged18+; admitted to a detention facility, expected to be released to Milwaukee County, WI who were willing to be tested for HIV; verbal communication in English
STT ILLINOIS			
OPT OUT	Cross-sectional study of opt-out HIV testing	Baseline only	Aged 18+; detained in the IL corrections system
CM JAIL	Non-randomized study of case-management intervention vs. standard of care	6, 12, 18 months	Aged 18+; HIV-infected; detained in IL corrections system (jail); expecting to reside in Chicago after release
CM PRISON	Non-randomized study of case-management intervention vs. standard of care	6, 12, 18 months	Aged 18+; HIV-infected; recently released from IL corrections system (prison); enrollment was within 60 days of release
START	1. RCT of CARE-Rapid (computer-assisted program) vs. TAU and 2. quasi-experimental study of manualized intervention vs. TAU, with follow-up at 3 months	3 months	Men aged 18+ with either unknown or believed negative HIV status within 90 days of release from NY detention center entering a residential substance abuse treatment program
STRIDE			
STRIDE 1	RCT of buprenorphine vs. placebo	Monthly for 12 months	Aged 18+; HIV-infected; meeting DSM-IV criteria for opioid dependence; resident of Washington, DC with eligibility for medical entitlements; English- or Spanish-speaking; no current opiate medications for chronic pain conditions or need to be placed on such medications; no current methadone doses over 30 mg/day, no AST and ALT >5× the ULN; no pregnancy or breast-feeding; no liver dysfunction; no suicidal ideation; no participation in pharmacotherapy trial in the previous 30 days
STRIDE 2	Longitudinal cohort study comparing treatment using opioid substitution therapy to no treatment	3, 6, 9, 12 months	Aged 18+; HIV-infected; meeting DSM-IV criteria for opioid dependence; resident of Washington, DC with eligibility for medical entitlements; English-speaking
SUCCESS	Non-randomized pilot study of Strengths-Based case management and texting	3, 12 months	Aged 18+; HIV-infected; detained or sentenced in jail or detention center and likely to leave within 6 weeks; no recent participation in randomized trial to improve retention in HIV care; English-speaking
UHS II			
UHS IIC	Cross-sectional study of HIV testing	Baseline only	Aged 18+; crack cocaine or injection drug use in the past 30 days
UHS IIL	Longitudinal cohort study of case management (Project Bridge) vs. usual treatment	Quarterly for 24 months	Aged 18+; HIV-infected; crack cocaine or injection drug use in the past 30 days

*ALT* alanine aminotransferase, *ART* antiretroviral therapy, *AST* aspartate aminotransferase, *CA* California, *CJ* criminal justice, *CT* Connecticut, *DC* District of Columbia, *DSM-IV* Diagnostic and Statistical Manual of Mental Disorders IV, *HCV* hepatitis C virus, *HIV* human immunodeficiency virus, *IL* Illinois, *MD* Maryland, *NC* North Carolina, *NY* New York, *RCT* randomized controlled trial, *RI* Rhode Island, *RNA* ribonucleic acid, *TAU* treatment as usual, *TX* Texas, *ULN* upper limit of normal, *WI* Wisconsin

^a^Study acronyms as in Table 1

The STTR CJ cohort includes 11,070 individuals with and without HIV who range in age from 18 to 77 years with a median baseline age of 37 years. The STTR CJ cohort is diverse and includes substantial numbers of African-American (64%) and Hispanic (12%) individuals as well as 17% percent women (Table 3). Given differences in enrollment criteria across STTR CJ cohort studies, the percentage reporting HIV risk behaviors varies considerably (Table 4). Injection drug use (IDU) ranged from 86% of participants reporting ever use in one study, with 64% in that study reporting IDU within the 30 days prior to incarceration, to very low levels in other studies (Table 4). Reports of recent condomless anal or vaginal intercourse also varied from 4 to 79% of participants. Recent use of marijuana, cocaine, and opioids, and binge drinking were common across all studies (Table 5) with 53% reporting use of multiple substances. Information regarding nonmedical prescriptions drug use is available from 9 of the studies and substudies. The participants were recruited from a wide range of CJ settings including jail, prison, and community supervision (pretrial, parole and probation) with particularly large numbers of individuals from jail, prison, and probation (Table 6).

**Table 3:** Demographic characteristics for STTR CJ cohort study participants

		Race/ethnicity, %									Self-identified gender, %				
Study^a^	N^b^	American Indian or Alaskan Native	Asian	Black or African American	Native Hawaiian or Other Pacific Islander	White	Hispanic or Latino	Some other race	Two or more races	Don’t know, refused, missing	Female	Male	Trans-gender	Don’t know, refused, missing	Age, median years (range)
BRIGHT															
BRIGHT 1	2405	2.4	0.0	51.6	0.0	28.0	12.8	1.5	3.6	0.1	17.8	82.1	0.2	0.0	39 (18-73)
BRIGHT 2	100	1.0	0.0	90.0	0.0	3.0	3.0	0.0	3.0	0.0	22.0	78.0	0.0	0.0	45 (28-70)
CARE+															
RCT	112	0.0	0.0	86.6	0.0	3.6	0.9	0.9	8.0	0.0	28.6	57.1	12.5	1.8	42 (19-63)
HCV RIDOC	250	4.8	1.2	14.8	0.0	46.8	22.4	2.0	8.0	0.0	6.4	93.6	0.0	0.0	31 (19-58)
HCV PROB	138	4.4	0.0	18.8	0.7	41.3	25.4	2.2	5.8	1.5	18.8	80.4	0.7	0.0	32 (19-68)
IMPACT	381	2.1	0.0	66.1	0.0	19.7	8.1	0.8	3.2	0.0	22.0	75.3	2.4	0.3	44 (20-64)
LINK LA	356	1.1	0.8	34.6	1.1	21.9	31.2	0.8	8.5	0.0	3.6	84.5	11.0	0.0	40 (21-69)
NEW HOPE	123	0.0	0.0	22.8	0.0	12.2	63.4	0.0	0.8	0.8	17.9	81.3	0.8	0.0	46 (21-67)
STT	3963	1.4	0.2	63.2	0.1	14.6	9.4	0.5	5.9	4.7	2.9	96.9	0.1	0.1	29 (18-73)
STT ILLINOIS															
OPT OUT	236	0.4	0.0	67.8	0.0	9.7	20.3	0.9	0.9	0.0	56.8	43.2	0.0	0.0	27 (18-60)
CM JAIL	376	1.1	0.3	81.4	0.0	7.7	9.0	0.0	0.5	0.0	17.6	79.8	2.7	0.0	43 (18-74)
CM PRISON	90	0.0	0.0	90.0	0.0	3.3	5.6	0.0	0.0	1.1	8.9	86.7	3.3	1.11	46 (19-64)
START	195	1.0	0.5	46.2	0.0	21.6	29.2	0.5	1.0	0.0	0.0	100	0.0	0.0	38 (19-65)
STRIDE															
STRIDE 1	50	0.0	0.0	90.0	0.0	0.0	2.0	0.0	4.0	4.0	28.0	68.0	2.0	2.0	53 (39-67)
STRIDE 2	109	0.0	0.0	96.3	0.0	1.8	0.9	0.0	0.0	0.9	46.8	45.9	7.3	0.0	51 (23-66)
SUCCESS	56	0.0	0.0	69.7	0.0	8.9	3.6	0.0	7.1	10.7	7.1	89.3	3.6	0.0	38 (21-58)
UHS II															
UHS IIC	2090	0.6	0.1	86.0	0.3	4.7	6.8	0.2	1.2	0.2	41.0	58.8	0.2	0.0	49 (18-77)
UHS IIL	48	2.1	0.0	83.3	0.0	4.2	6.3	0.0	4.2	0.0	29.2	60.4	10.4	0.0	47 (27-66)

CJ, criminal justice; STTR, seek, test, treat and retain

^a^Study acronyms as in Table 1

^b^There are 33 participants who are included in more than one sub-study: 3 in BRIGHT 1 and BRIGHT 2, and 30 in UHSIIC and UHSIIL

**Table 4:** Risk behavior characteristics for STTR CJ cohort study participants

		Injection drug use, %^c^				Shared injection equipment, %^c^		Condomless sex, %^c^	
Study^a^	N^b^	Ever use: yes	Ever use: don’t know, refused, missing	Recent use: yes	Recent: don’t know, refused, missing	Recent use	Recent use: don’t know, refused, missing	Recent	Recent: don’t know, refused, missing
BRIGHT									
BRIGHT 1	2405	22.4	0.04	4.3	12.3	1.1	12.3	49.9	12.7
BRIGHT 2	100	51.0		10.0		2.0		16.0	1
CARE+									
RCT	112	14.3	1.8	4.5	6.3	3.6	5.4	78.6	3.6
HCV RIDOC	250	17.6		10.0		4.4		−	
HCV PROB	138	13.0		4.4		1.5		−	
IMPACT	381	−		−		−		−	
LINK LA	356	−		−		−		54.8	3.7
NEW HOPE	123	86.2	3.3	64.2	3.3	17.9	4.9	25.2	3.3
STT	3963	10.5	0.8	6.1	1.2	3.9	0.9	49.1	10.8
STT ILLINOIS									
OPT OUT	236	−		7.6		−		−	
CM JAIL	376	−		8.0	0.3	2.7	0.3	28.5	2.7
CM PRISON	90	−		8.9	1.1	3.3	1.1	38.9	1.1
START	195	−		3.6		−		25.1	0.5
STRIDE									
STRIDE 1	50	76.0	4.0	34.0	4.0	4.0	8.0	4.0	6.0
STRIDE 2	109	49.5		15.6		3.7	0.9	17.4	6.4
SUCCESS	56	−		10.7		−		51.8	3.6
UHS II									
UHS IIC	2090	43.0		26.5		−		64.5	0.05
UHS IIL	48	37.5		22.9				52.1	

*CJ* criminal justice, *STTR* seek, test, treat and retain

^a^Study acronyms as in Table 1

^b^There are 33 participants who are included in more than one sub-study: 3 in BRIGHT 1 and BRIGHT 2, and 30 in UHSIIC and UHSIIL

^c^Reference periods differed across studies; 30 days: STRIDE 1, NEWHOPE, START, SUCCESS, 90 days: BRIGHT 1 and 2, STRIDE 2, all STT Illinois studies, CARE + HCV RIDOC, CARE + RCT, STT, 180 days: LINK LA, UHS II. The following studies measured pre-incarceration risk behaviors: CARE+ RCT, CARE+ HCV RIDOC, LINK LA, NEW HOPE, STT, STT ILLINOIS OPT OUT, STT ILLINOIS CM JAIL, and STT ILLINOIS CM PRISON

**Table 5:** Substance use distribution for STTR CJ study participants

		Alcohol use, %^c,e^		Substance use, %^d,e^					Multiple substance use, %^d,e^
Study^a^	N^b^	Any	Binge	Marijuana	Cocaine	Opioids	Stimulants	Other	≥2 substances (including alcohol)
BRIGHT									
BRIGHT 1	2405	48.2	28.7	27.5	10.7	13.0	0.3	2.9	29.9
BRIGHT 2	100	44.0	25.0	22.0	26.0	23.0	1.0	3.0	38.0
CARE+									
RCT	112	80.4	60.7	35.7	37.5	17.0	8.9	20.5	54.5
HCV RIDOC	250	71.6	60.4	60.8	24.8	31.2	9.2	22.0	66.8
HCV PROB	138	63.8	37.0	42.0	15.9	10.9	2.9	4.4	39.1
IMPACT	−	−	−	−	−	−	−	−	−
LINK LA	356	53.1	24.7	54.2	17.1	10.1	58.1	9.8	66.3
NEW HOPE	123	37.4	22.8	23.6	77.2	91.9	0.8	8.9	81.3
STT	3963	46.5	31.4	37.7	14.8	13.8	9.8	14.4	38.9
STT ILLINOIS									
OPT OUT	−	−	−	−	−	−	−	−	−
CM JAIL	376	71.0	49.5	45.0	39.1	32.5	14.1	10.4	65.7
CM PRISON	90	75.6	53.3	63.3	44.4	41.1	13.3	13.3	77.8
START	195	72.8	53.9	35.9	21.5	21.0	1.0	12.3	47.2
STRIDE									
STRIDE 1	50	58.0	22.0	6.0	24.0	92.0	0.0	6.0	58.0
STRIDE 2	109	62.4	33.0	24.8	41.3	83.5	6.4	11.0	69.7
SUCCESS	56	71.4	48.2	51.8	48.2	25.0	21.4	1.8	57.1
UHS II									
UHS IIC	2090	79.7	54.6	61.7	90.8	47.3	10.6	12.0	92.3
UHS IIL	48	64.6	39.6	66.7	66.7	22.9	6.3	8.3	72.9

*CJ* criminal justice, *STTR* seek, test, treat and retain

^a^Study acronyms as in Table 1

^b^There are 33 participants who are included in more than one study: 3 in BRIGHT 1 and BRIGHT 2, and 30 in UHSIIC and UHSIIL

^c^Alcohol reference periods differed across studies; 30 days: LINK LA, NEW HOPE, START; UHS II; 90 days: BRIGHT 1 and 2, STT; 180 days: STT Illinois; 1 year: CARE + RCT, SUCCESS; not specified: STRIDE 1 and 2, START

^d^Substance reference periods differed across studies; 30 days: LINK LA, NEW HOPE, START Together; STRIDE 1, SUCCESS, UHS II; 90 days: BRIGHT 1 and 2, CARE+ RCT and HCV RIDOC, STT Strategies, STRIDE 2; 180 days: STT Illinois

^e^The following studies measured pre-incarceration alcohol and substance use: CARE+ RCT, CARE+ RIDOC, LINK LA, NEW HOPE, STT, STT JAIL, STT PRISON, and SUCCESS

**Table 6:** Supervision status for STTR CJ cohort study participants

		CJ supervision status, %							
Study^a^	N^b^	No current CJ supervision	Jail	Prison	Probation	Parole	Probation/parole	Other	Don’t know, refused, missing
BRIGHT									
BRIGHT 1	2405	0	0	0	81.3	15.2	3.5	0	0.08
BRIGHT 2	100	0	0	0	69.0	25.0	6.0	0	0
CARE+									
RCT	−	−	−	−	−	−	−	−	−
HCV RIDOC	250	0	0	100	0	0	0	0	0
HCV PROB	138	0	0	0	97.8	0.7	0.7	0	0.7
IMPACT	381	0	0	100	0	0	0	0	0
LINK LA	356	0	100	0	0	0	0	0	0
NEW HOPE	123	0.0	58.5	27.6	3.3	0.8	0.0	5.7	4.1
STT	3963	0	100	0	0	0	0	0	0
STT ILLINOIS									
OPT OUT	236	0	100	0	0	0	0	0	0
CM JAIL	376	0	100	0	0	0	0	0	0
CM PRISON	90	14.4	0.0	0.0	0.0	78.9	4.4	1.1	1.1
START	−	−	−	−	−	−	−	−	−
STRIDE									
STRIDE 1	50	44.0	0.0	0.0	16.0	30.0	4.0	4.0	2.0
STRIDE 2	109	78.9	0.0	0.0	3.7	10.1	0.0	6.4	0.9
SUCCESS	56	0	100	0	0	0	0	0	0
UHS II									
UHS IIC	2090	68.0	0	0	23.5	6.1	2.1	0	0.2
UHS IIL	48	52.1	0	0	35.4	4.2	8.3	0	0

*CJ* criminal justice, *STTR* seek, test, treat and retain

^a^Study acronyms as in Table 1.

^b^There are 33 participants who are included in more than one study: 3 in BRIGHT 1 and BRIGHT 2, and 30 in UHSIIC and UHSIIL

### Harmonization results

By combining data across multiple studies, we include individuals involved with varying aspects of the CJ system, enabling comparisons that cannot typically be done within individual studies. For example, harmonizing data enables analyses involving subgroups such as transgender individuals and other subgroups that are often too small to assess in individual studies.

A key finding from our conduct of data harmonization is the need for multiple approaches. One technique involved modern psychometric approaches such as item response theory (IRT) to co-calibrate scales. Co-calibration enables people from studies who responded to different instruments to have scores that are “calibrated together” on a single common metric. IRT provides a suite of tools to model and account for measurement precision. We use this approach to address differences in the depression and anxiety measures across studies. In contrast, we found a different approach was needed to harmonize domains like ART adherence, which are behavioral rather than latent traits. Below we describe two harmonization efforts for ART adherence and risk behaviors.

### Harmonization of ART adherence measures

We used CJ studies that measured ART adherence with both the AIDS Clinical Trials Group (ACTG) questionnaire and the Visual Analogue Scale (VAS) measure to develop rules and a formula to co-calibrate both instruments. By co-calibrating adherence items from the ACTG (7 day recall) with the VAS, we were able to harmonize studies having different approaches to measuring adherence [31]. We found that 44% of PLWH in the STTR CJ cohort who were on ART reported adherence rates <95% for the prior 30 days and 38% reported having missed one or more doses in the prior 14 days. Furthermore, we found important variations not only in ART adherence in different groups, but also in how adherence measures work. The findings also highlighted problems with the VAS as a single item adherence measure and showed its validity varied across groups. These results would not have been feasible within single studies but instead were possible by harmonizing multiple studies to gain both necessary sample size and variation [32].

### HIV sexual risk behavior harmonization

Eight studies administered a standardized risk behavior assessment tool to evaluate sexual risk behaviors in these reference periods: 30 or 90 days prior to incarceration for jail and prison detainees, or the previous 30 or 90 days for participants enrolled in the community. We described sexual risk behaviors of HIV-infected and uninfected participants in these studies and compared men and women [33, 34]. To test for differences in risk behaviors between men and women, we performed individual patient data meta-analysis to combine information across studies. Findings indicated a high prevalence of condomless anal or vaginal sex among PLWH and uninfected participants, particularly women with HIV. For example, the adjusted odds ratio for condomless sex was 1.84 (1.16-2.95) for women with HIV compared with men with HIV [34]. By aggregating data across studies, we were able to overcome the much lower prevalence of women in CJ populations and, in turn, in each of the individual CJ studies.

## Discussion

STTR CJ is a unique cohort that combines data from 11 studies that together address steps across the HIV treatment cascade focusing on participants with CJ involvement. The STTR CJ cohort includes a large and diverse assemblage of individuals who are at risk for, or living with HIV. The pooled data cohort is a sample of men, women, and transgender individuals across different racial/ethnic groups in multiple CJ settings (prison, jail, pre-trial, community supervision) as well as participants with no CJ involvement. The intervention studies span the HIV treatment cascade and have broad geographic locations across the US with participants enrolled from 11 states and the District of Columbia. These characteristics provide the opportunity to address questions of great importance for HIV prevention and treatment particularly related to individuals involved in the CJ system, those at risk or with HIV and using substances, and the unique risks and clinical needs of specific subgroups. For example, we are particularly interested in understanding the impact of changes in the intensity of substance use on HIV care cascade outcomes such as viral suppression and adherence, the impact of incarceration and release on the HIV care continuum, and to better understand risk behaviors of individuals upon release from incarceration. Finally, the study investigators and DCC have expertise in clinical, epidemiologic, CJ, biostatistical, and data harmonization areas. This team is well-positioned to address trans-disciplinary scientific questions and identify new questions that will arise as HIV care and prevention continues to evolve, and we have developed harmonized measures to allow for combining studies to answer these questions.

There are limitations to the STTR CJ cohort data. Some of these limitations are inherent in combining data from independent studies with different enrollment criteria and different study designs. While the STTR CJ cohort includes a large number of CJ-involved PLWH or persons at risk of HIV, this combination of studies is not a representative sample of those involved in the CJ system in the US, and in particular may underrepresent rural areas. Harmonization of some variables has been difficult due to modifications made by some individual studies to standardized instruments and data collection timeframes. For example, some studies modified timeframes for behaviors that were assessed (30 days vs. 90 days) to fit their study follow-up periods. Missing data varied by participating study and some studies have limited or no data on certain domains. This means that it is not feasible to use all studies in some analyses. For example, studies varied in whether they focused on current substance use or included type of substances at the time of incarceration. HIV status also varied with studies focused on later stages in the HIV cascade more likely focusing on PLWH. Similarly, studies that focused on late stages of the cascade cannot contribute to HIV risk analyses in that all participants are PLWH. The heterogeneity of study designs in the participating studies including both observational and interventional studies makes it feasible to study all the different stages of the STTR cascade but also complicates approaches to combining studies. When merging these studies it is critical to identify the subset of the STTR CJ cohort appropriate for and able to provide data on the research questions of interest. These studies recruited from diverse source populations. For example, two studies recruited participants who were at high risk of CJ system involvement but not necessarily already involved with the CJ system at baseline. This variation prevents the naïve pooling of studies, as baseline characteristics are different between studies, and requires clustering of participants by study be accounted for using a mixed model or generalized estimating equation approach. Longitudinal follow-up duration and frequency varies with several studies having limited follow-up time although we will continue to include all additional data collected over follow-up, so long as studies remain ongoing. While this variation limits power for analyses looking at longitudinal outcomes, the STTR CJ cohort remains better powered than individual studies.

The STTR CJ cohort is designed to allow expansion for new studies and domains as needed to address important questions in the HIV research agenda particularly related to the STTR treatment cascade. New studies will be added as needed to increase demographic, geographic, or clinical diversity and broaden scientific expertise. The STTR CJ cohort welcomes collaboration from interested parties and has policies to ensure multi-center analytic research proposals are developed and results analyzed collaboratively and fairly.

## Conclusions

This cohort provides a large study sample across different CJ settings (jail, prison, community supervision) and includes both persons with and without HIV, many of whom engage in illicit drug use. Compared to single studies, the combined data of the CJ cohort provides increased statistical power to determine the public health benefit of the STTR paradigm, address research questions on specific groups at risk of exiting the treatment cascade, and improve understanding of the intersection of drug use and HIV treatment.

## Abbreviations

ACTG: AIDS Clinical Trials Group
ART: Antiretroviral therapy
CJ: Criminal Justice
DCC: Data Coordination Center
IDU: Injection drug use
IRT: item response theory
NIDA: National Institute on Drug Abuse
NIMH: National Institute of Mental Health
OAR: Office of AIDS Research
STTR: Seek, Test, Treat and Retain
US: United States
VAS: Visual analogue scale
VL: Viral load
